# Are housing tenure and car access still associated with health? A repeat cross-sectional study of UK adults over a 13-year period

**DOI:** 10.1136/bmjopen-2016-012268

**Published:** 2016-11-02

**Authors:** A Ellaway, L Macdonald, A Kearns

**Affiliations:** 1 MRC/CSO Social and Public Health Sciences Unit, University of Glasgow, Glasgow, UK; 2 Department of Urban Studies, University of Glasgow, Glasgow, UK

**Keywords:** MENTAL HEALTH, car ownership, housing tenure

## Abstract

**Background:**

It is usually assumed that housing tenure and car access are associated with health simply because they are acting as markers for social class or income and wealth. However, previous studies conducted in the late 1990s found that these household assets were associated with health independently of social class and income. Here, we set out to examine if this is still the case.

**Methods:**

We use data from our 2010 postal survey of a random sample of adults (n=2092) in 8 local authority areas in the West of Scotland. Self-reported health measures included limiting longstanding illness (LLSI), general health over the last year and the Hospital Anxiety and Depression Scale.

**Results:**

We found a statistically significant relationship between housing tenure and all 4 health measures, regardless of the inclusion of social class or income as controls. Compared with owner occupiers, social renters were more likely to report ill-health (controlling for social class—LLSI OR: 3.24, general health OR: 2.82, anxiety η^2^: 0.031, depression η^2^: 0.048, controlling for income—LLSI OR: 3.28, general health OR: 2.82, anxiety η^2^: 0.033, depression η^2^: 0.057) (p<0.001 for all models). Car ownership was independently associated with depression and anxiety, with non-owners at higher risk of both (controlling for income—anxiety η^2^: 0.010, depression η^2^: 0.023, controlling for social class—anxiety η^2^: 0.013, depression η^2^: 0.033) (p<0.001 for all models).

**Conclusions:**

Our results show that housing tenure and car ownership are still associated with health, after taking known correlates (age, sex, social class, income) into account. Further research is required to unpack some of the features of these household assets such as the quality of the dwelling and access to and use of different forms of transport to determine what health benefits or disbenefits they may be associated with in different contexts.

**Strengths and limitations of this study**

- Our study showed that home and car ownership are still associated with better health after the 2008 economic downturn.
- We conducted a repeat cross-sectional study of a random sample of residents of the same West of Scotland areas 13 years apart (1997 and 2010), using a similar postal questionnaire and thus were able to examine potential change.
- Health selection may have affected our findings in that people with poor health may be more likely to live in social rented housing as they have priority.
- In this cross-sectional study, we were unable to examine the direction of causation; for example, existing poor health might affect income or employment and thus influence ability to buy a home or a car.

## Introduction

A number of studies have shown that housing tenure and car access are associated with health,^1–6^ and they have often been viewed as indicators or proxies of social class, income or wealth rather than having any direct relationship with health.^7^ However, in studies conducted in the late 1990s, we showed that these assets were associated with health even after taking individual characteristics such as social class, income, age and sex into account.^8^ ^9^ Studies conducted elsewhere broadly supported our findings.^10^ ^11^ However, since the late 1990s, the onset of the global economic downturn in 2008 has led to a slowdown in the UK housing market (a drop from 76% homeownership in 2001 to 65% in 2010^12^) and in other countries such as the USA where home ownership has traditionally been the preferred tenure.^13^ A number of studies have shown that the economic downturn was associated with a rise in mental health problems^14–16^ often associated with mortgage arrears.^17^ ^18^ The drop in price and increase in housing repossessions (sixfold increase in the UK between 2004 and 2009^12^ with similar increases in the USA^19^) raise questions of whether ‘the bloom is off the rose of homeownership’.^20^ There has also been a drop in car sales (new car registrations in the UK halved between 1998 and 2009, dropping from 230 000 in 1998 to 110 000 in 2009^21^) and rising running costs.^22^ These changes therefore raise questions over whether we would still find similar results to those of our studies in the late 1990s; perhaps owner-occupied homes and cars are not seen as so beneficial or as a cultural requirement following the global economic downturn and the uncertainty since. In this paper, we therefore examine whether home and car ownership are still associated with better health, and we explore these patterns separately for social class and income as these two measures have different patterns of consumption.^23^

## Methods

In 2010, we repeated our 1997 postal survey of a random stratified sample of adults in eight local authority areas in the West of Scotland. THAW 2010 was based on THAW 1997, a study designed to examine three objectives, first, the statistical associations between long-term morbidity and mental health and well-being on the one hand, and housing tenure and car ownership on the other (while controlling for sociodemographic and psychological characteristics); second, the role of housing quality, residential environment and use of cars, in influencing illness and psychological health; and third, the meaning of housing tenure and car ownership in people's daily lives.^9^ ^24–30^

THAW 2010 draws on respondents from the same geographical areas to our 1997 postal survey and uses a very similar postal questionnaire to the previous study. The survey included standard questions on the respondents' mental and physical health and well-being, lifestyle, housing, neighbourhood, transport, employment and finance. The majority of the items in the questionnaire were based on self-complete items used in previously conducted studies such as the West of Scotland Twenty-07: Health in the Community study.^31^ Our 1997 questionnaire was piloted with 200 individuals and adjustments made to the questionnaire thereafter.

In our 2010 survey, we decided to replicate our study in the same West of Scotland areas as our 1997 study due to its socially heterogeneous composition. As with our 1997 survey, our random sample of the general population was stratified to reduce selection bias^32^ using a geodemographic classification of neighbourhood type (using ACORN, Scottish version^33^) to ensure that all types of residential neighbourhoods (ranging from ‘affluent consumers in large houses’ to ‘poorest council estates’) were included in correct proportions.

The postal questionnaire (see online supplementary file), with three reminders (using Dillman's total design method^34^), was sent out in the autumn of 2010. We achieved a response rate of 38% (2092 completed questionnaires), from a sample of 5521 adults drawn from the electoral roll in the 8 local authority areas which make up the Glasgow and Clyde Valley Structure Plan area in the West of Scotland. The estimated population in this area in 2010 was 1 763 430, and contains marked variations in social status and in health.^35^ Survey respondents' ages ranged from 17 to 95 years. The sociodemographic characteristics of THAW 2010 were comparable to the previous THAW 1997 study; for example, respondents' own social class was similar in THAW 1997 and THAW 2010 (65% and 70% in the non-manual social class groups, respectively). Compared with the West Central Scotland population, our achieved study sample characteristics were broadly similar for sex and for age; 56% were female, and 65% were of working age (18–60 years), compared with 52% and 62%, respectively, within West Central Scotland.^36^ Within our sample, 85% of respondents had access to at least one car or van, while within the 2010 Scottish Household Survey, within West Central Scotland, 70% had access to a car (does not include van access).^37^

10.1136/bmjopen-2016-012268.supp1 supplementary file

THAW 2010 was approved by the Ethics Committee of the Faculty of Law, Business and Social Sciences at the University of Glasgow. Here, we examine four domains of self-assessed health, three of which are similar to our previous paper:^9^ chronic, recent and mental health problems and health in general, measured, respectively, by the presence/absence of limiting longstanding illness (LLSI), perceived health over the past year as either excellent/good or fair/poor and the seven-item depression subscale of the Hospital Anxiety and Depression Scale ((HADS), higher scores on HADS indicate greater reported symptoms).^38^ The suite of self-assessed health measures used in the 1997 study was identified from the literature.^8^ ^9^ ^30^ ^39^ In the present paper, we also examined the HADS anxiety seven-item subscale as we have previously shown that some aspects of the home are associated with anxiety among social rented respondents,^39^ but there is a possibility that home ownership since the economic downturn may be also associated with anxiety. Social class was based on own occupation, using registrar general's sixfold classification,^40^ income was equivalised household income (ie, adjusted for family composition).^41^

We excluded respondents (n=101) who reported they were economically inactive because of permanent sickness or disability (to reduce the possibility of reverse causation). We also excluded respondents who could not be categorised as owner occupier or social renter, that is, those who were privately renting (N=58) or those who lived in a hostel, a tied home or in relation/partner's home (N=14). We excluded respondents with missing data (assumed missing at random) for independent, dependent or control variables, within each model.

Logistic regression was used to explore relationships between housing tenure and car access, and LLSI and general health, each in three ways: unadjusted; adjusted for age, sex and marital status; and adjusted for age, sex, marital status and income or social class. Generalised linear modelling was used to investigate equivalent models for anxiety and depression scores using η^2^>values (η^2^ represents effect sizes, ie, the proportion of variance associated with main effects from ANOVA;^42^ effect sizes are considered ‘small’ if η^2^>0.01, ‘medium’ if η^2^ 0.06 and ‘large’ if η^2^>0.14^43^). Within the models, we also investigated various interactions between tenure or car access and sex; and tenure or car access and marital status, particularly as the latter differed between owners and social renters (see table 1).

**Table 1:** Characteristics by tenure status

	Owner occupier (n=1104)	Social renter (n=115)
*Sample characteristics*		
Sex (%)		
Males	44.7	47.0
Females	55.3	53.0
Age		
Mean (minimum–maximum)	51.4 (17–91)	52.0 (20–90)
Social class (%)		
I/II Professional, managerial and technical	50.1	20.0
III Skilled non-manual	25.0	24.3
III Skilled manual	12.8	19.1
IV/V Partly skilled and unskilled	12.1	36.5
Marital status (%)		
Living with significant other	72.0	40.9
Not living with significant other	28.0	59.1
*Material assets*		
Car ownership (%)		
Owner	88.7	58.3
Non-owner	11.3	41.7
*Health measures*		
Limiting longstanding illness (%)		
Has limiting longstanding illness	44.6	78.6
No limiting longstanding illness	55.4	21.4
General health (%)		
Excellent/good	79.3	47.8
Fair/poor	20.7	52.2
*HADS anxiety		
Mean (minimum–maximum)	6.1 (0–21)	8.3 (0–18)
*HADS depression		
Mean (minimum–maximum)	3.4 (0–21)	5.8 (0–21)

*Clinical case >10

## Results

### Housing tenure

We found a statistically significant relationship between all four health measures and tenure, regardless of the inclusion of social class or income as controls (see table 2). For LLSI, the relationship was attenuated by including age, sex, marital status and social class (unadjusted OR: 3.46, adjusted OR: 3.24) or income (unadjusted OR: 3.95, adjusted: 3.28) but remained significant (p<0.001 for all models). This was also the case for tenure and general health, controlling for the sociodemographic variables, including social class (unadjusted OR: 3.40, adjusted OR: 2.82) or income (unadjusted OR: 4.37, adjusted OR: 2.82) (p<0.001 for all models). The relationship between tenure and HADS anxiety was slightly strengthened by the inclusion of control variables and social class (unadjusted η^2^: 0.030, adjusted η^2^: 0.031), or income (unadjusted η^2^: 0.030, adjusted η^2^: 0.033) (p<0.001 for all models). Similarly with tenure and HADS depression, controlling for sociodemographic variables including social class (unadjusted η^2^: 0.046, adjusted η^2^: 0.048), or income (unadjusted η^2^: 0.053, adjusted η^2^: 0.057) (p<0.001 for all models).

**Table 2.** Odds and η2 for the relation between health measures and (a) tenure and (b) car access; unadjusted, and adjusted for age, sex and marital status, and for age, sex, marital status and social class or income.

	LLSI		Poor/Fair general health		Depression		Anxiety	
	Odds	Sig.	Odds	Sig.	η2	Sig.	η2	Sig.
**(a) Social Rented Tenure (owner occupier as reference)**								
*Social Class*								
Unadjusted model	3.46	0.001	3.40	0.001	0.046	0.001	0.030	0.001
Adjusted for age, sex, marital status	3.50	0.001	3.17	0.001	0.047	0.001	0.031	0.001
Adjusted for age, sex, marital status, social class	3.24	0.001	2.82	0.001	0.048	0.001	0.031	0.001
Number	1523		1528		1530		1530	
***Income***								
Unadjusted model	3.95	0.001	4.37	0.001	0.053	0.001	0.030	0.001
Adjusted for age, sex, marital status	3.82	0.001	4.07	0.001	*0.055	0.001	0.032	0.001
Adjusted for age, sex, marital status, income	3.28	0.001	2.82	0.001	*0.057	0.001	0.033	0.001
Number	1305		1315		1316		1315	
**(b) No Car access (access to car as reference)**								
*Social Class*								
Unadjusted	1.97	0.001	2.03	0.001	0.022	0.001	0.009	0.001
Adjusted for age, sex, marital status	1.38	0.068	1.50	0.014	0.022	0.001	0.010	0.001
Adjusted for age, sex, marital status, social class	1.27	0.189	1.36	0.072	0.023	0.001	0.010	0.001
Number	1586		1591		1593		1593	
***Income***								
Unadjusted model	2.03	0.001	2.16	0.001	0.031	0.001	0.012	0.001
Adjusted for age, sex, marital status	1.38	0.084	1.59	0.008	0.031	0.001	0.013	0.001
Adjusted for age, sex, marital status, income	1.16	0.435	1.17	0.384	0.033	0.001	0.013	0.001
Number	1362		1371		1372		1371	

*significant interactions between tenure and sex, and tenure and marital status – see tables 3 and 4.

### Car access

When controlling for age, sex, marital status and social class, the relationship between car access and LLSI is no longer statistically significant (unadjusted OR: 1.97 (p<0.001), adjusted OR: 1.27 (p=0.189)) (see table 2). This was also true when controlling for income (unadjusted OR: 2.03 (p<0.001), adjusted OR: 1.16 (p=0.435)). Similar results were found for car access and general health when controlling for age, sex, marital status and social class (unadjusted OR: 2.03 (p<0.001), adjusted OR: 1.36 (p=0.072)), or income (unadjusted OR: 2.16 (p<0.001), adjusted OR: 1.17 (p=0.384)). On the other hand, the relationship between car access and anxiety was only slightly stronger by the inclusion of the control variables, and social class (unadjusted η^2^: 0.009, adjusted η^2^: 0.010), or income (unadjusted η^2^: 0.012, adjusted η^2^: 0.013) (p<0.001 for all models) as controls (see table 2). Additionally, this was the case for car access and depression, when controlling for social class (unadjusted η^2^: 0.022, adjusted η^2^: 0.023) or income (unadjusted η^2^: 0.031, adjusted η^2^: 0.033) (p<0.001 for all models).

### Interactions

For the income-adjusted depression and tenure model, a significant interaction between tenure and sex was found (p=0.013); there was a significant difference in the mean depression scores between male owner occupiers (3.88) and male social renters (4.48), and an almost twofold difference between female owner occupiers (3.63) and female social renters (5.98). In both cases, social renters had higher depression scores, but the difference in scores between female tenure groups (p<0.001, η^2^=0.086) is greater than that of the males (p<0.001, η^2^=0.029) (table 3). A significant interaction between tenure and marital status also exists (p=0.014). Social renters have higher depression scores than owner occupiers, but the difference was 50% greater in the case of single people (owner: 4.16, renter: 6.13, p<0.001, η^2^=0.089) than in the case of those living with a significant other (owner: 3.31, renter 4.28, p<0.001, η^2^=0.016) (table 4).

**Table 3:** Tenure and depression (sex × tenure) (controls: age, and income)

	Mean depression score
Male	
Owner occupier (523)	3.88
Social renter (65)	4.48
	(sig.=0.001, η^2^=0.029)
Female	
Owner occupier (651)	3.63
Social renter (77)	5.98
	(sig.=0.001, η^2^=0.086)

**Table 4:** Tenure and depression (marital status×tenure) (controls: age, sex, and income)

	Mean depression score
Single	
Owner occupier (332)	4.16
Social renter (85)	6.13
	(sig.=0.001, η^2^=0.089)
Lives with sig. other	
Owner occupier (842)	3.31
Social renter (57)	4.28
	(sig.=0.001, η^2^=0.016)

## Discussion

In our 2010 study of a random sample of adults in the West of Scotland, we found that household assets such as owning one's home or car are still associated with some of the health we observed in our earlier study in the late 1990s. Other studies broadly support our findings;^44^ and a study of national health surveys for 10 European countries found that housing tenure was associated with better health in some countries (Great Britain and the Netherlands) but not all, suggesting that the meaning and importance of tenure is context-specific.^45^ The importance of context is reflected in an Australian study which found that while mental health varied by tenure, home ownership was no longer associated with better health once other sociodemographic characteristics were taken into account.^46^

There are several plausible reasons why housing tenure might be associated with the health outcomes we have examined. Social housing in the UK is frequently provided in the form of estates which often have associations with particular, historical forms of employment which affect long-term health. Estates are also considered to have distinct cultures^47^ and established norms, etc, which may affect health behaviours (and in turn physical health) and psychosocial health. Behaviours on social housing estates can also manifest as antisocial behaviour which causes residents a great deal of anxiety, so that concerns about disturbance, potential threats and safety could underlie some of the greater anxiety felt by social renters. The Scottish Household Survey has consistently found that social renters report various types of antisocial behaviour problems 2–3 times more often than owner occupiers, and that those people living in the most deprived areas feel far less safe in their neighbourhood than others.^48^

There are two possible reasons why social renting might be associated with higher levels of depression. First, environmental quality (eg, street cleanliness) is often worse in social housing areas, due to poor design as well as lower levels of environmental maintenance by service providers relative to need in deprived areas.^49^ Other research with residents in such areas has shown a strong association between neighbourhood quality (eg, litter and graffiti) and mental well-being.^50^ Second, relative deprivation^51^ may play a role, so that a psychosocial pathway operates between inequality and mental health^52^ in that those people without assets such as their own homes or cars feel disadvantaged in a status-oriented society,^53^ which may in turn be reflected in higher depression scores among social renters and non-car owners. Past research has shown associations between the perceived relative quality of the home and self-esteem^25^ and that mental well-being is higher where people feel they live in desirable homes and in neighbourhoods that people rate highly.^54^ It is of interest to note that our findings that home ownership is associated with better health were in turn patterned by the route to home ownership as the differences between tenure type and health were smaller among those who had bought their homes under the Right to Buy (RTB) scheme (data not shown). The RTB scheme is a UK policy whereby public sector tenants had the right to purchase their homes at heavily discounted prices. The scheme ended on 31 July 2016 in Scotland but continues elsewhere in the UK. Many be of the dwellings purchased under this scheme tended to of better quality and in more popular social housing areas, leading to residualisation whereby the majority of housing stock left for social renting was of poorer quality and in less popular neighbourhoods.^55^ It is also of interest to note that other studies have found that by including that housing wealth (assessed by house value) may shed more light on the possible mechanisms linking tenure and health than tenure alone (see, eg, Connolly *et al*'s^56^ study in Northern Ireland which linked census data on health with objective data on house values obtained from the national assessor).

Similar psychosocial mechanisms may operate in respect of cars, and again we would expect the effects to be less than those of housing tenure, as shown in the depression and anxiety results reported here. Interestingly, the association between car access and these psychosocial measures is stronger than in our 1997 survey; this may reflect the growing importance of car ownership for psychosocial health and everyday life^57^ since the late 1990s.^58^ ^59^ The association between housing tenure and LLSI when controlling for social class was also stronger (an almost twofold increase in the odds of reporting) in our 2010 survey than our 1997 study. This may be a reflection of the composition of our achieved sample in 2010 as it contained a larger proportion of older adults than our 1997 sample, Although we controlled for age in our models, it may be that other unmeasured variables may play a part.

We studied residents of the same areas 13 years apart (1997 and 2010) using a similar questionnaire and thus were able to examine potential change after the economic downturn. In accord with downward trends in survey participation^60–62^ and response rates in deprived areas,^63^ we achieved a lower response rate (38%) compared with our 1997 study (50%). However, the response rate for each question, considered another measure of the survey's response rate,^64^ was at least 95% for more than 90% of the 78-item questionnaire. Our sample comprises a largely urban sample and it may be that we would have found different results in a more rural population such as the highlands of Scotland^65^ and rural areas elsewhere in the UK^66^ ^67^ where car ownership is a necessity. Our findings that housing tenure and car ownership are associated with health may be subject to residual confounding, in that there are likely to be unmeasured socioeconomic circumstances that affect these associations, particularly those measured across the life course.^68^ Health selection may also play a part in that individuals in poor health may be ‘sorted’ into social housing due to the UK priority points system.^69^ Moreover, in an attempt to reduce reverse causation in our analysis, we excluded those respondents who reported they were economically inactive because of permanent sickness or disability; however, it is still possible that poor health lowers earnings and in turn the likelihood of being able to buy a home and/or a car. However, in a cross-sectional study like ours, it is not possible to disentangle these factors.

In conclusion, given our findings that housing tenure and car ownership are still independently associated with health, after taking known correlates such as age, social class, income and gender into account, it still seems therefore that it is important to unpack some of the features of these household assets, such as the quality of the dwelling and access to and use of different forms of transport, to determine what health benefits or disbenefits they may currently be associated with in the UK and in different contexts.
